# Association between Hypotension, Low Ejection Fraction and Cognitive Performance in Cardiac Patients

**DOI:** 10.3233/BEN-2009-0261

**Published:** 2010-06-10

**Authors:** Rebecca F. Gottesman, Maura A. Grega, Maryanne M. Bailey, Scott L. Zeger, William A. Baumgartner, Guy M. McKhann, Ola A. Selnes

**Affiliations:** ^1^Department of NeurologyJohns Hopkins University School of MedicineBaltimoreMDUSA; ^2^Department of Cardiac SurgeryJohns Hopkins University School of MedicineBaltimoreMDUSA; ^3^Johns Hopkins Bloomberg School of Public HealthJohns Hopkins UniversityBaltimoreMDUSA; ^4^The Zanvyl-Krieger Mind Brain InstituteJohns Hopkins UniversityBaltimoreMDUSA

**Keywords:** Heart failure, cognition, blood pressure, brain ischemia

## Abstract

*Background and Purpose:* Impaired cardiac function can adversely affect the brain via decreased perfusion. The purpose of this study was to determine if cardiac ejection fraction (EF) is associated with cognitive performance, and whether this is modified by low blood pressure.

*Methods:* Neuropsychological testing evaluating multiple cognitive domains, measurement of mean arterial pressure (MAP), and measurement of EF were performed in 234 individuals with coronary artery disease. The association between level of EF and performance within each cognitive domain was explored, as was the interaction between low MAP and EF.

*Results:* Adjusted global cognitive performance, as well as performance in visuoconstruction and motor speed, was significantly directly associated with cardiac EF. This relationship was not entirely linear, with a steeper association between EF and cognition at lower levels of EF than at higher levels. Patients with low EF and low MAP at the time of testing had worse cognitive performance than either of these alone, particularly for the global and motor speed cognitive scores.

*Conclusions:* Low EF may be associated with worse cognitive performance, particularly among individuals with low MAP and for cognitive domains typically associated with vascular cognitive impairment. Further care should be paid to hypotension in the setting of heart failure, as this may exacerbate cerebral hypoperfusion.

## 1. Introduction

Impaired cardiac function can adversely impact the brain either via embolization, leading to stroke [1], or via hypoperfusion. One potential consequence of hypoperfusion is impaired cognitive performance, secondary to transient alterations in cerebral blood flow [2], or even direct structural brain injury (from stroke or perhaps white matter injury).

Reversible adverse cerebral effects of heart failure are supported by reports of individuals undergoing cardiac transplantation who have post-transplant improvement in cognition [3] and improvement in cerebral vascular flow velocities (measured by transcranial Doppler) [4]. In contrast, more permanent structural injury is supported by the recent finding of higher total white matter hyperintensity volume on brain MRI among individuals with congestive heart failure (CHF), compared to controls without impaired cardiac function [5]. Furthermore, in one autopsy study of 23 individuals who had prior heart transplants, 70% had histopathologic evidence of brain injury, with vascular lesions in 60%. The pattern of these lesions was con-

ISSN 0953-4180/09,10/$17.50 © 2009/2010 – IOS Press and the authors. All rights reserved sistent with hypoperfusion, impaired cerebral autoregulation and hypotension [6]. Thus, it is likely that heart failure is associated with some combination of transient (and reversible) effects and permanent structural abnormalities.

Although *higher* blood pressure is associated with worse outcomes in the general population, *low* blood pressure in CHF is associated with worse survival [7], with an estimated 13% increase in mortality per every 10 mm Hg drop in systolic blood pressure among CHF patients [8]. Hypotension may interact with hypoperfusion from impaired cardiac pumping ability in patients with CHF, and may be particularly detrimental to cognition. Hypotension (defined as systolic blood pressure $< 130$ mm Hg) is more common among hospitalized individuals with both CHF and cognitive impairment (46%), compared to those with CHF and with no cognitive impairment (27%), or to individuals without CHF (32–33%) [9]. Thus, it is possible that the combination of transient mechanisms of hypoperfusion, such as from hypotension, and chronic impaired cardiac pumping ability, might act in synergy to worsen cognitive performance. The purpose of this study was to explore the association between ejection fraction (EF), the percentage of blood pumped out of the heart with each heart beat, and cognitive performance, among individuals with vascular disease but not acutely unstable with congestive heart failure symptoms. Specifically, we hypothesized that the *combination* of low EF and low blood pressure would be associated with worse cognitive performance.

## 2. Methods

### *2.1. Patient inclusion*

From 1997–2001, 326 patients were enrolled as part of an ongoing, controlled, observational study of cognitive outcomes after coronary artery bypass graft (CABG) surgery. This study was approved by the Johns Hopkins IRB committee, and all patients completed written informed consent at baseline enrollment. Inclusion criteria required that patients be able to undergo neuropsychological testing, able to give written informed consent, able to sit upright, were native English speaking, and not mechanically ventilated. Patients were not excluded for medical reasons. For this analysis, individuals with baseline echocardiography or cardiac catheterization were included. Participants included individuals from 3 groups: (1) preoperative on-pump CABG surgery; (2) preoperative off-pump CABG surgery; and (3) individuals with diagnosed coronary artery disease receiving medical therapy. A fourth group, individuals without any vascular disease ("heart-healthy controls") also underwent the same cognitive assessment at baseline (see below). This was not a randomized study and details about methods are described elsewhere [10]. Although the study was a 6-year longitudinal study, for the purposes of this analysis, only cross-sectional cognitive data from each participants' baseline, prior to surgery (or at enrollment in the study, among individuals in the medical group), was used. Cognitive trajectories over the duration of the study, or after surgery for individuals in groups 1 and 2, were not examined for this analysis.

### *2.2. Measurement of Ejection Fraction (EF)*

Baseline EF data was recorded at entry into the study. Because it was obtained as part of clinical practice, in some cases it was obtained from echocardiography (either trans-thoracic or trans-esophageal), although in most cases it was obtained from cardiac catheterization. Normal EF is considered $> 55\%$. EF was analyzed both as a continuous variable and categorically, using clinically relevant delineations: $\geqslant 55\%$, 35–55%, and $\leqslant 35\%$.

### *2.3. Cognitive testing*

All individuals underwent baseline cognitive assessment, including tests in multiple cognitive domains. Methods have been reported elsewhere [11]. An age, sex, and education-adjusted Z score was calculated for each individual in each of 8 cognitive domains (Table 1), and a global Z score (mean of the 8 cognitive domain scores) was calculated. Z scores were calculated based on performance of the "heart healthy" group, whose mean scores were given a Z score of 0. For the Trails A and B and Grooved Pegboard tests, scores were truncated at 240 seconds.

### *2.4. Other data collection*

Blood pressure (BP) was measured at the time of the cognitive testing visit, within 5–10 minutes prior to the start of the cognitive testing. Mean arterial pressure was calculated (2/3 Diastolic BP + 1/3 Systolic BP). In addition, baseline demographic and medical history information was collected at the time of recruitment in all individuals.

Table 1
Cognitive tests performed within each of 8 cognitive domains

| Cognitive domain | Test/ sub-tests included in each domain |
|---|---|
| Verbal Memory | Rey Auditory Verbal Learning Test (RAVLT): Total score |
| | RAVLT: Delayed recall |
| | RAVLT: Retention |
| | RAVLT: Recognition |
| Visual Memory | Rey complex figure: Retention |
| | Rey complex figure: Delayed recall |
| Visuoconstruction | Rey complex figure: Copy |
| | Block design |
| Language | Boston naming test |
| Motor speed | Grooved pegboard dominant hand |
| | Grooved pegboard nondominant hand |
| Psychomotor speed | Trail Making Test – Part A |
| | Written alphabet |
| Attention | RAVLT trial #1 |
| | Mini-mental state examination: Serial 7's subtest |
| Executive function | Trail Making Test- Part B |

### *2.5. Statistical analysis*

Stata version 10.1 for Macintosh was used for statistical analyses [12]. Basic descriptive statistics were used. For univariate associations based on level of EF (3 categories, described above), Fisher's exact tests were used for categorical variables, and linear regression was used for continuous variables. As described above, age, sex, and education-adjusted Z scores were calculated globally, and within each domain, based on performance relative to the heart healthy control group. Linear regressions were performed, with EF as the primary independent variable and score on each cognitive domain as the dependent variable, in separate analyses. These analyses were repeated both with EF as a continuous value, both as a linear and nonlinear (cubic spline) predictor, and with EF categorized into the 3 categories as described above.

Linear regression models were also repeated including adjustment for "stroke probability score" [13], which is calculated based on age, prior stroke history, hypertension, and diabetes.

For analyses of the interaction between blood pressure and EF, an interaction term was created for mean arterial pressure (MAP, divided into quartiles, with the lowest quartile used for the interaction analysis) X EF (continuous, and categorical, in separate models), and this interaction term was used as a covariate in a regression of cognitive performance score, added to the model described above, for each separate cognitive test. Lowess techniques were used to generate curves to show the relationship between EF and cognition depending on MAP level.

## 3. Results

### *3.1. Study population*

There were 234 individuals with EF data that were included in this analysis. Normal EF was classified in 47.0%, with moderately reduced EF in 35.9% and low EF in 17.1%. Characteristics of these three groups are shown in Table 2. Individuals with low EF did have significantly lower blood pressure than individuals in the other groups. Of individuals with EF in the markedly reduced range ($\leqslant$ 35%), 36% had a MAP, at the start of testing, in the lowest quartile, compared to 21% of the moderately reduced EF group, and 21% of the normal EF group ($p = 0.08$). This was independent of the use of beta-blockers (used by 63% of the sample); an individual's MAP at the onset of testing averaged 1.7 points lower for every 10 percentage points lower EF ($p = 0.009$), with no change in results when use of beta-blockers was included as a covariate. A total of 14 individuals had both EF in the markedly reduced range and a MAP in the lowest quartile at the start of testing.

### *3.2. Ejection fraction and cognition*

Table 3 shows the results of the primary regression analyses. When analyzed continuously (Table 3), both linearly and nonlinearly, ejection fraction significantly predicted Z-score in the global cognitive domain, as well as motor speed and visuoconstruction. Analysis of EF using a cubic spline generally led to steeper direct associations between EF and cognition across lower EF values, with flat or less steep associations at higher values of EF. The utility of analyzing EF in a nonlinear

Table 2
Demographics, baseline information on individuals in 3 categories of ejection fraction

| Variable | Normal EF ($\geqslant$ 55%) ($N = 110$) | Moderately reduced EF (35–55%) ($N = 84$) | Markedly reduced EF ($\leqslant$ 35%) ($N = 40$) | p-value |
|---|---|---|---|---|
| Age | 64.4 | 63.1 | 64.8 | 0.57 |
| Sex (% Male) | 71.8% | 80.0% | 72.5% | 0.42 |
| Surgical study group | | | | 0.15 |
| On-pump CABG | 60.9% | 66.7% | 65.0% | |
| Off-pump CABG | 19.1% | 16.7% | 30.0% | |
| Vascular nonsurgical control | 20.0% | 16.7% | 5.0% | |
| Race (% Black) | 8.2% | 8.3% | 12.5% | 0.69 |
| Level of education | | | | 0.38 |
| $<$ 12 yrs | 18.2% | 21.4% | 22.5% | |
| 12–16 yrs | 50.9% | 54.8% | 62.5% | |
| 16+ years | 30.9% | 23.8% | 15.0% | |
| History of past stroke | 3.6% | 8.3% | 2.5% | 0.24 |
| Mean arterial pressure (mm Hg) | 93.6 | 96.4 | 86.0 | 0.0004 |
| Systolic blood pressure (mm Hg) | 133.3 | 137.2 | 122.6 | 0.005 |
| Diastolic blood pressure (mm Hg) | 73.7 | 76.0 | 79.5 | 0.001 |

Table 3
Regression coefficients for each of 8 cognitive domains. The coefficient in the first column represent the change in the age, sex, and education-adjusted Z-score for each 10% increase in cardiac ejection fraction (EF), with EF analyzed continuously as a linear predictor

| | Analysis with EF as linear predictor | | Analysis with EF as nonlinear predictor | | |
|---|---|---|---|---|---|
| | Beta | p-value | Beta (below knot) | Beta (above knot) | p-value |
| Global | 0.11 | 0.04 | 0.03 | −0.02 | 0.04 |
| Motor | 0.15 | 0.02 | 0.02 | −0.003 | 0.05 |
| Executive function | 0.07 | 0.14 | 0.02 | −0.01 | 0.15 |
| Attention | −0.001 | 0.98 | −0.004 | 0.004 | 0.94 |
| Visual memory | 0.07 | 0.14 | 0.02 | −0.01 | 0.18 |
| Verbal memory | 0.05 | 0.29 | 0.02 | −0.01 | 0.23 |
| Visuoconstruction | 0.13 | 0.03 | 0.03 | −0.02 | 0.02 |
| Language | 0.04 | 0.48 | 0.03 | −0.03 | 0.11 |
| Psych | 0.06 | 0.24 | 0.007 | −0.001 | 0.50 |

manner is demonstrated in the following figures: Fig. 1 shows the actual data, and the linear relationship shown by the data. Figure 2 shows the predicted cubic spline, with a steeper association between EF and Z-score at lower EF values.

When analyses were repeated excluding the single outlier (Fig. 1; EF reported at 98%) because of concerns that this might be erroneous (or at the very least might represent hyperdynamic left ventricular function associated with diastolic cardiac dysfunction), the associations between each of the domains and EF became even stronger, with slightly larger effect sizes and lower p-values.

In more fully adjusted models, with adjustment for stroke probability score, the strength of the associations became larger, with more significant p-values, for the global (Beta $= 0.013$, $p = 0.02$, visuoconstruction (Beta $= 0.016$, $p = 0.01$), and motor speed domains (Beta $= 0.014$, $p = 0.02$) (the other domains remained nonsignificant; these values are for EF as a continuous linear predictor, with similar results for EF as a cubic spline). This covariate was not used in the primary models, however, because of missing stroke probability data in 8 individuals.

### 3.3. *Analyses of MAP and EF in predicting cognitive performance*

Table 4 shows the domains for which a significant ($p < 0.05$) association was found between EF and Z-score, in which we tested for an interaction between MAP quartile and EF level. The regression coefficients for EF were higher (consistent with a steeper relationship) for individuals with lower MAP values than for those with higher MAP values, although individual p-values for interaction were not significant.

This association is further demonstrated in Fig. 3, where the nonparametrically generated lowess curves for individuals with low MAP (in the lowest quartile) are shown along with those for individuals with normal MAP, for the motor and global domain, where these associations were the most apparent. Results are very

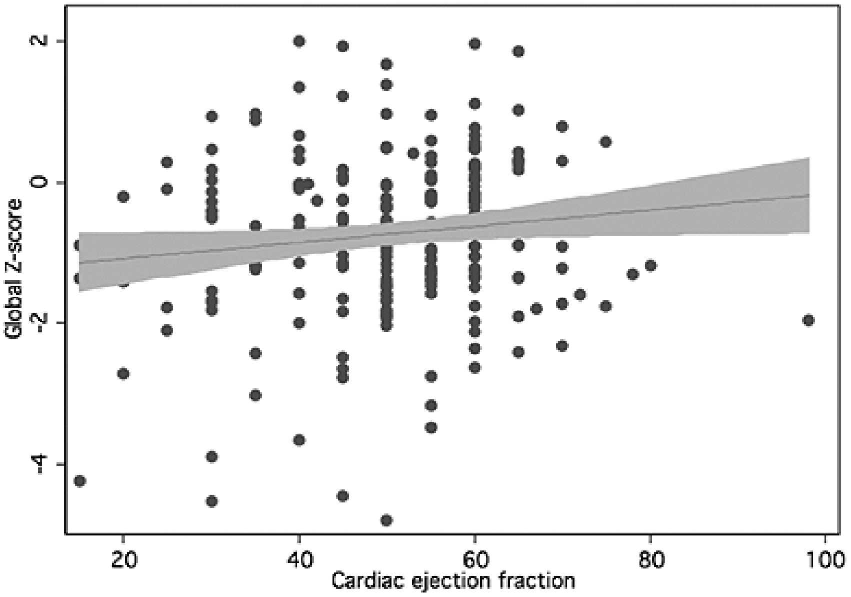

Fig. 1. Linear plot of relationship between Z-score and cardiac EF. Actual data points, linear fit, and 95% confidence bands are shown.

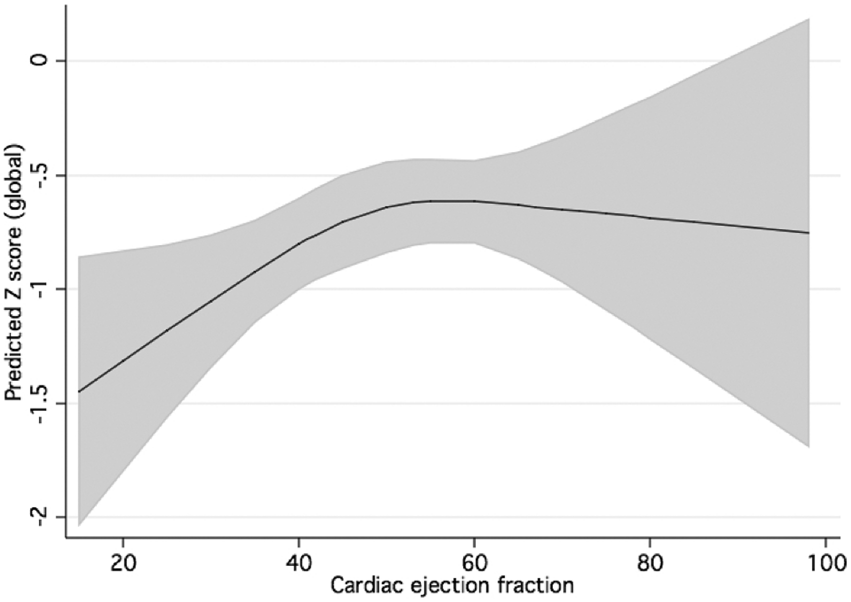

Fig. 2. Cubic spline demonstrating association between global cognition Z-score and cardiac EF. The predicted association with 95% confidence bands are shown.

similar when MAP from immediately before cognitive testing is averaged with the value from immediately after cognitive testing.

### 3.4. Contribution of the study group

Although analysis included baseline cross-sectional data only, and thus would not be affected by whether individuals were scheduled to get surgery or not, reduced EF was differentially (but non-significantly) distributed among individuals in the three study groups (on-pump, off-pump, and other vascular controls). 19% of the 196 individuals scheduled to have surgery (either on- or off-pump) versus only 5% of the 38 vascular controls had EF in the markedly reduced range ($p = 0.07$). Adjust-

Table 4
Interaction between *lowest* MAP quartile and EF (per 10% increase in EF) in the prediction of cognitive Z-score. Regression coefficients and their individual p-values are shown for the association between EF and Z-score for individuals with MAP in the lowest, versus in any other quartile (columns 1 and 2)

| | Coefficient if MAP in lowest quartile | Coefficient if MAP not in lowest quartile | p-interaction |
|---|---|---|---|
| Global Z score | 0.27 ($p = 0.03$) | 0.05 ($p = 0.38$) | 0.10 |
| Motor | 0.26 ($p = 0.04$) | 0.10 ($p = 0.19$) | 0.28 |
| Visuoconstruction | 0.22 ($p = 0.02$) | 0.11 ($p = 0.14$) | 0.42 |

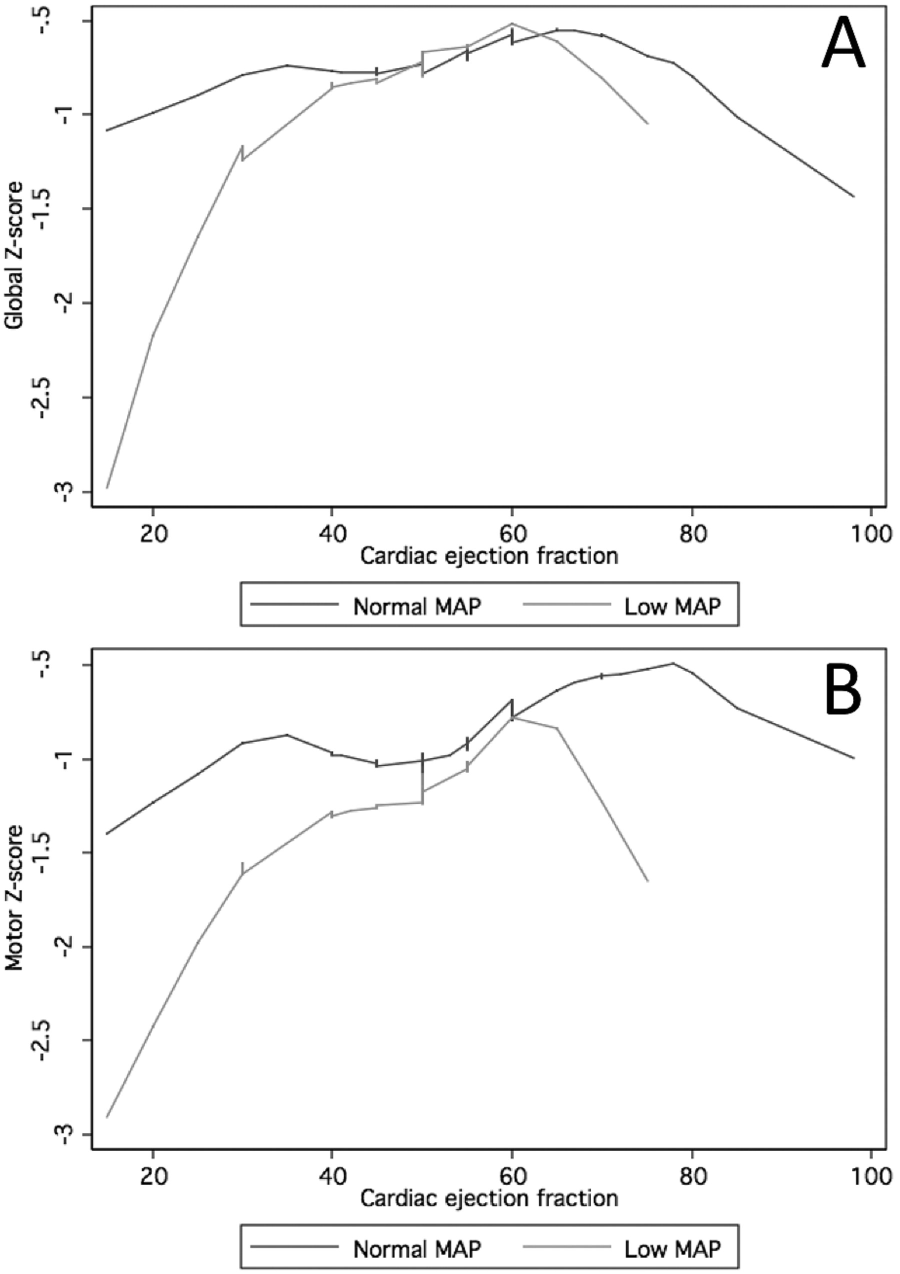

Fig. 3. Lowess curves for individuals with normal mean arterial pressure (MAP) (top 3 quartiles) versus low MAP (lowest quartile), demonstrating association between global (panel A) and motor (panel B) Z-scores and cardiac ejection fraction.

ment for study group attenuated some, but not all, of the associations reported above (the primary analyses remained significant).

### *3.5. Characteristics of individuals with missing EF data*

Because EF data was only available on close to two-thirds of the entire sample of vascular patients in the overall study, we compared characteristics of these participants to those with missing EF data. Patients with missing data were older (mean age 66.8 years, vs. 64.0 years, $p = 0.02$) than those with EF measured. Otherwise, both groups had similar percentages of women (26.1% vs. 25.2%, $p = 0.87$), and had similar performances across all of the cognitive tests.

## 4. Discussion

This analysis has demonstrated that cognitive performance was associated with level of ejection fraction among individuals with vascular disease, but without specific clinical evidence of heart failure. Worse performance was found in individuals with EF in the low range (below 35%), both globally and in the cognitive domains often implicated in vascular cognitive impairment, motor speed and visuoconstruction. EF appeared to have a nonlinear association with cognition, particularly the global measure, with the largest decrement in performance associated with further decreases in EF at lower values. This relationship appeared to be even stronger in the presence of hypotension, although the interaction terms were not statistically significant.

Our results are supported by other studies describing an association between impaired cardiac pumping ability and impaired cognitive performance, both in individuals with symptomatic [14–16] and asymptomatic heart failure [17]. In addition, other investigators have shown that the extent of cardiac dysfunction is associated with degree of cognitive dysfunction, primarily in clinical heart failure populations. In one study, cognitive difficulties were more frequent in 31 CHF patients, compared with 31 controls with coronary artery disease, and were positively associated with worse ejection fraction (EF) and cardiac output (CO), both indicators of the pumping ability of the heart [18]. Despite this body of evidence, however, two systematic reviews of case-control studies found no level I evidence supporting a relationship between CHF and cognitive impairment [19,20].

In other studies, the pattern of cognitive impairment in patients with CHF particularly involves memory [16] as well as "global" cognitive functioning, attention, psychomotor slowing and impaired executive functioning [20,21]. This pattern is similar, although not identical, to the findings we report in this paper. In general, however, involvement of memory and motor/psychomotor speed might be expected with a combination of subcortical disease of any cause and hippocampal ischemia, from hypoperfusion. In laboratory models of transient global cerebral ischemia (e.g. in cardiac arrest), the regions of the brain most vulnerable to ischemia include the CA1 layer of the hippocampus [22], leading to a common amnestic syndrome after clinical exposure to global hypoxic-ischemic injury, such as during cardiac arrest [23]. Furthermore, mice exposed to chronic cerebral hypoperfusion develop problems with working memory [24].

Further support for a role of hypoperfusion in the etiology of cognitive problems in CHF patients comes from transcranial Doppler imaging data, with lower cerebral blood flow reported in patients with decompensated CHF [2]. Evidence that this hypoperfusion may be transient, and may not lead to persistent structural abnormalities (such as stroke or white matter injury) is found in studies of individuals awaiting heart transplantation [25] who have improvement in cognitive performance post-transplant. In addition, our findings of a trend towards an interaction between blood pressure and EF further support a role of hypoperfusion in the development of cognitive dysfunction in CHF patients.

In previous studies, the specific role of hypotension among patients with impaired cardiac function has not been clear. Although probability of cognitive dysfunction has been more likely in CHF patients with lower systolic blood pressure [9] the exact nature of this relationship has been unclear. Our data suggest that transient fluctuations in blood pressure may have important consequences for cognitive functioning of individuals with CHF. Although hypotension is more common in patients with severe heart failure, it is possible that certain medications may contribute adversely to cerebral function, by lowering blood pressure to levels that are not optimal for the brain. We found no evidence that our findings were due to use of concurrent medications, but this warrants further evaluation with more detailed medication information.

Our study is primarily limited by the relatively modest sample size and lack of data on other heart failure symptoms or other cardiac function measures. Although these participants did not have acute heart failure symptoms, the wide range of EF would be better interpreted if more precise descriptions of clinical symptoms were available. Similarly, other cardiac measures, such as cardiac output, might be more useful in associations with cognitive function than EF. In addition, we were limited by the fact that two separate methods to obtain EF were used, depending on what was available in a given patient. Previous studies suggest that EF measured by multiple techniques (including echocardiography and contrast cineangiography, as used in this study) has high correlation but only moderate agreement [26].

Other confounders could also exist that could explain some of the association between cognition and EF, such as other vascular diseases or vascular risk factors. By repeating the analyses including adjustment for the "stroke probability score", which is a value derived based on presence/ absence of prior stroke, age group, hypertension, and diabetes, we accounted for major confounding from other vascular disease, but there could be other diseases or characteristics about those particular diseases which could impact both cognition and EF. Depression is another potential confounder, as this has been associated with heart failure, and with cognitive impairment. Repeated analyses adjusting for depressive symptoms led to slight attenuation of effect sizes and level of significance, but did not reveal any large changes in the coefficients or level of significance for associations between cardiac function and the cognitive tests.

The strengths of this study include the consistency of the associations for a number of tests, in different cognitive domains, with particular involvement in those domains typically affected in vascular cognitive impairment. Our finding that EF and cognition may not have a completely linear relationship will be important in further studies, and demonstrates that even a small change in EF at a low range may make a large difference in cognitive function. Our study is further strengthened by the ability to analyze blood pressure at the time of testing, as many CHF patients have fluctuations in their blood pressure and possibly in their cognitive functioning. The finding on the synergy between blood pressure and cardiac function is novel, and may ultimately lead to changes in clinical care.

Our results support the presence of lower cognitive performance among individuals with impaired cardiac function, with a dose-response type of relationship between cardiac and cognitive function, with worse performance in individuals with low ejection fraction category. In addition, the suggestion of an interaction between blood pressure at the time of testing and EF provide further support to the theory that hypoperfusion to the brain may occur in individuals with CHF, and that this may be multifactorial. Further study is needed to determine the longitudinal nature of cognitive performance among individuals with CHF and the role of fluctuations in blood pressure along that longitudinal course.

## Funding

This study was funded by the NIH (RO1-NS035610 (GM)) and the Dana Foundation (GM). The authors have no conflicts of interest to disclose.

## Disclosure

The authors have no conflicts of interest to disclose.

## Acknowledgements

The authors thank Pamela Talalay for her editorial assistance.
